# Functional Morphology of the Cardiac Jelly in the Tubular Heart of Vertebrate Embryos

**DOI:** 10.3390/jcdd6010012

**Published:** 2019-02-27

**Authors:** Jörg Männer, Talat Mesud Yelbuz

**Affiliations:** 1 Group Cardio-Embryology, Institute of Anatomy and Embryology UMG, Georg-August-University Goettingen, D-37075 Goettingen, Germany; 2 Department of Cardiac Sciences, King Abdulaziz Cardiac Center, Section of Pediatric Cardiology, King Abdulaziz Medical City, Ministry of National Guard Health Affairs, Riyadh 11426, Saudi Arabia; yelbuzta@ngha.med.sa

**Keywords:** embryonic heart tube, extracellular matrix, cardiac jelly, hydraulic skeleton, heart skeleton, valveless pumping, blood flow, non-circular cross sections, ballooning, trabeculation

## Abstract

The early embryonic heart is a multi-layered tube consisting of (1) an outer myocardial tube; (2) an inner endocardial tube; and (3) an extracellular matrix layer interposed between the myocardium and endocardium, called “cardiac jelly” (CJ). During the past decades, research on CJ has mainly focused on its molecular and cellular biological aspects. This review focuses on the morphological and biomechanical aspects of CJ. Special attention is given to (1) the spatial distribution and fiber architecture of CJ; (2) the morphological dynamics of CJ during the cardiac cycle; and (3) the removal/remodeling of CJ during advanced heart looping stages, which leads to the formation of ventricular trabeculations and endocardial cushions. CJ acts as a hydraulic skeleton, displaying striking structural and functional similarities with the mesoglea of jellyfish. CJ not only represents a filler substance, facilitating end-systolic occlusion of the embryonic heart lumen. Its elastic components antagonize the systolic deformations of the heart wall and thereby power the refilling phase of the ventricular tube. Non-uniform spatial distribution of CJ generates non-circular cross sections of the opened endocardial tube (initially elliptic, later deltoid), which seem to be advantageous for valveless pumping. Endocardial cushions/ridges are cellularized remnants of non-removed CJ.

## 1. Introduction

**Figure 1:**
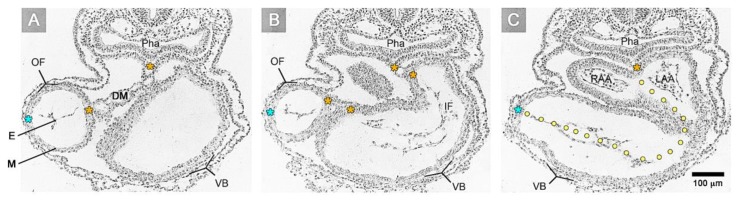
These transverse histological sections of a human embryo with 10 pairs of somites (Carnegie stage 10) show the inner architecture of an S-shaped heart tube as seen in routine histological preparations. The heart was fixed in a systolic state so that its lumen is partially obliterated. (**A**) Section at the level of the proximal outflow (= OF) of the ventricular tube. (**B**) Section of at the level of the inflow (= IF) of the ventricular tube. (**C**) Section at the level of the developing–right and left atrial appendages (= RAA, LAA). Note that the myocardium (= M) and endocardium (= E) are separated by an “empty” space, called the myoendocardial space. Note also: (1) The difference in the thickness of the myoendocardial space between the developing atria and ventricles; and (2) the paired character of the myoendocardial space, which reflects the origin of the heart from bilaterally paired heart fields. The dorsal mesocardium (= DM, and indicated by orange asterisks) and the obliterated endocardial tube can be used as anatomical landmarks for the original midsagittal fusion plane of the left- and right-sided heart fields (marked by the dotted line in section C). Light blue asterisks mark the original ventral midline of the heart tube. Further abbreviations: VB = ventricular bend; Pha = pharynx/foregut.

The heart is the first organ to form and function in vertebrate embryos. During the initial phase of its pumping activity, the vertebrate heart has the design of a tubular conduit that generates unidirectional blood flow by a valveless pumping mechanism [1]. The wall of this tubular heart has a relatively simple architecture. It consists of only two different epithelial layers, from which one forms the outer envelope and the other forms the inner lining of the tube. The outer envelope of the heart tube, which is in contact with the pericardial fluid, is formed by a two-layered epithelium of embryonic cardiomyocytes, nowadays called the “primary myocardium” [2]. The inner lining of the heart tube, which is in contact with the blood, is formed by a single-layered endothelium called the primitive or “primary endocardium”. The myo- and endocardial layers of the embryonic heart tube are not in direct contact with each other. Instead, a wide, cell-free space is found between them (Figure 1). 

In the tradition of the Department of Embryology of the Carnegie Institution, this space was named the “myoendocardial space” [3,4,5,6,7]. During the 19th and early 20th century, the structure and material properties of the substance filling the myoendocardial space were deduced only from light microscopical observations on routine histological sections (Figure 1), which showed either an “empty” space or a space filled with traces of “filamentous” material. Based on such morphological data, the content of the myoendocardial space was interpreted either as serous fluid [8,9] or as a cell-free, gelatinous material [10,11,12]. Based on in vivo observations on chick embryonic hearts, Carl L. Davis was able to confirm the gelatinous character of the transparent material filling the myoendocardial space and named it the “cardiac jelly” [13]. His work, which has been published in the form of a meeting abstract in 1924, may be regarded as an early landmark in research on the extracellular matrix (ECM) of the early embryonic heart, since the term, “cardiac jelly” (CJ), found its way into the “Terminologia Embryologica” produced by the “Federative International Committee on Anatomical Terminology” [14]. Today, the term, “cardiac jelly” (CJ), is frequently used synonymously for the myoendocardial space of the early embryonic heart tube.

**Figure 2:**
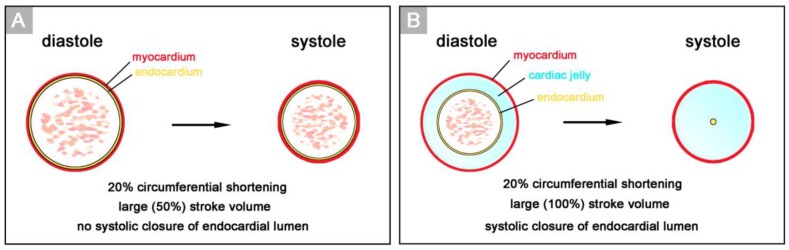
These schematic drawings illustrate Barry’s geometrical analyses on cross sections of hypothetical heart tubes of a large diameter. (**A**) In a hypothetical heart tube without CJ, physiological shortening of the contracting myocardium (20%) produces a large change (50%) in the cross sectional area of the lumen (= stroke volume), but does not close the lumen. (**B**) In a hypothetical heart tube with CJ, physiological shortening of the contracting myocardium will produce a large change (100%) in the stroke volume as well as complete closure of the endocardial lumen, if the thickness of the CJ layer is about 45% of the radius of the diastolic lumen. Note that the spatial distribution of CJ in the hypothetical heart tube does not correspond to the non-uniform distribution of CJ (bilaterally paired character) found in real embryonic heart tubes (see Figure 1A).

Classical embryology did not pay much attention to the CJ and other forms of embryonic ECM [15]. Therefore, its structure, composition, and functions remained obscure for a long time following the confirmation of its gelatinous character. The first study on the function of CJ was published in 1948 [16]. In this pioneering work, Alexander Barry related the presence of a thick myoendocardial layer of viscoelastic CJ to biophysical functions, namely the mechanical support of valveless pumping. He used geometric calculations to show that the presence of a relatively thick layer of CJ is required for complete end-systolic occlusion of the lumen of vertebrate embryonic heart tubes (Figure 2). This was thought to improve the efficiency of the pump action of the valveless heart tube by preventing the backflow of blood.

Barry, furthermore, has speculated that the diastolic reopening of the lumen of the embryonic heart tube is powered by passive energy stored in the elastic deformation of the CJ during systole. This mechanism was said to explain the diastolic sucking action of heart tubes, which he observed in experiments on living chick embryos. Barry’s ideas about the role of the CJ in the normal pumping function of valveless embryonic heart tubes are supported by the phenotype of CJ-deficient mouse embryos, which show signs of severe heart insufficiency during the initial phase of embryonic heart action [17].

In the years following Barry’s pioneering work, there was little interest in uncovering further biophysical functions of the CJ. The research has mainly focused on the biochemical composition of the CJ, its dynamic changes during development, and the cell biological effects of CJ components, as reviewed by [18]. Research on organ-scale biophysical functions of the CJ and its structural background had a short revival in the late 1970s and early 1980s. Manasek and coworkers have shown that the hydration of the CJ generates a turgor pressure that, together with a network of delicate CJ fibers, stabilizes the shape of the outer myocardial tube in a manner comparable to an inflated balloon [19,20]. These data indicate that the CJ behaves as a “hydraulic skeleton” and, therefore, may be regarded as the first skeleton of the vertebrate heart (for reviews about hydraulic skeletons see [21,22,23]). Manasek and coworkers have, furthermore, speculated that hydraulic expansion of the CJ, together with unisotropic arrangement of fibrils within the myocardial envelope, may drive the deformation of the initially straight embryonic heart tube into a helically coiled heart loop [19,20,24]. The idea of CJ-driven heart looping, however, was disproved by the observation that experimental removal of CJ at pre-looping stages did not prevent cardiac looping in mouse and chick embryos [25,26].

We should note here that the phenotype of a jelly-like ECM is not specific for the early embryonic heart. It merely represents the generalized phenotype of early embryonic ECMs [11,27]. Thus, CJ is only a local variant of a group of embryonic ECMs that may be termed “embryonic jellies”. The high hydration of embryonic jellies generates turgor pressures that, together with extracellular fiber systems and epithelial walls, contribute to mechanical stabilization of the shape of the early embryo and some of its organs [11,27]. Therefore, besides several other currently known functions, embryonic jellies act as hydraulic skeletons.

This short phase of revived interest in the organ-scale biophysics of CJ was followed by a relatively long phase of remarkable progress in our understanding of the molecular and cellular biological aspects of the CJ. The continuous progress in knowledge about the functional significance of the early cardiac ECM was documented in several excellent review articles, which appeared over the last 20 years [28,29,30,31]. While reading these papers, we have noted, to our surprise, that the contributions of the CJ to the pumping mechanics of the embryonic heart tube were either completely ignored [28,30] or were reduced to the cryptic statement that CJ helps in the “regulation of blood flow” [29,31]. Comments on the significance of the CJ for the mechanical pump action of the early embryonic heart tube are also missed in contemporary monographs and textbooks on cardiac development [32,33]. The proper pumping function of the tubular embryonic heart is crucial for the survival of higher vertebrate embryos as well as for correct morphogenesis of their hearts [34,35]. Thus, a deep understanding of the morphogenesis of congenital heart defects needs an understanding of the ontogeny of the cardiac pumping function. This, in turn, will remain fragmentary without knowledge about the organ-scale mechanics of the CJ and its structural base.

This review article is intended to close the above-mentioned gap of knowledge found in contemporary reviews about the ECM of the early embryonic heart. Our paper, therefore, focuses exclusively on the currently neglected morphological and biomechanical aspects of CJ. We hope that this article will contribute to a better understanding of the morphogenesis and pumping function of the early embryonic heart of vertebrates.

## 2. Definition of Terms and Chronology of CJ Development 

Davis introduced the term, “cardiac jelly“ (CJ), for characterization of the cell-free, gelatinous substance filling the myoendocardial space of the embryonic heart tube [13]. In this review article, we define CJ in this strict sense. According to this definition, the ECM of the early embryonic heart will be called CJ if it fulfills two requirements: (1) The ECM must occupy an anatomically well-demarcated myoendocardial space that is bordered by the basal laminae of the primary myocardium and endocardium; and (2) the ECM has to be free of cells.

These two conditions are present together only during a relatively narrow time span of early cardiogenesis. This period starts with the first appearance of an anatomically well-demarcated myoendocardial space and ends in a region-specific manner either with the invasion of endocardium-derived mesenchymal cells into the CJ (atrio-ventricular canal and outflow portion of the heart tube) or with the formation of a trabeculated myocardial wall (ventricular bend of the heart tube). In chick embryos, this period covers the developmental stages 10 to 16/17, according to Hamburger and Hamilton (HH-stages) [36]; in mice, it covers somite-stages 4 to 12 (embryonic days 8 and 9); and in human embryos, it covers Carnegie stages 10 and 11.

**Figure 3:**
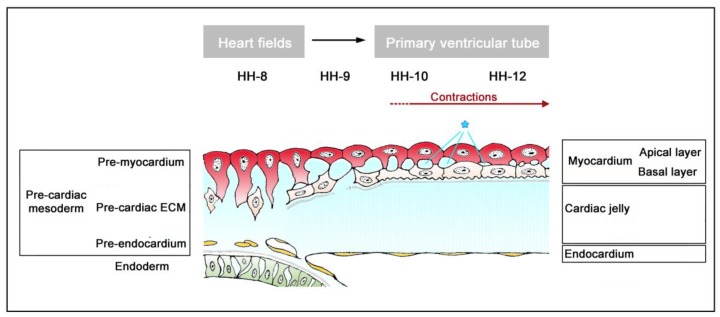
This schematic drawing illustrates the establishment of the primary wall architecture and initiation of myocardial contractions in the tubular hearts of higher vertebrate embryos as seen in the chick (based on data from Manasek [37] and Sakai et al. [39]). Developmental stages according to Hamburger and Hamilton [36] are indicated as HH-8, etc. The components of the primary heart wall derive from the pre-cardiac mesoderms. These parts of the lateral plate mesoderm harbor: (1) A coelomic epithelium formed by myocardial progenitors (pre-myocardium); (2) endocardial progenitors (pre-endocardium), which are found in close association with the endoderm; and (3) a gelatinous ECM (pre-cardiac ECM), which connects the pre-myocardium and pre-endocardium with each other, and with the endoderm. Note that the pre-cardiac mesoderms do not possess an anatomically well-demarcated myoendocardial space. The appearance of such a space depends on myocardial differentiation and the formation of endocardium-lined tubes. During myocardial differentiation, the discontinuous basal layer of the pre-myocardium becomes transformed into a continuous cell sheet bordered by a basal lamina. Consequently, the developing myocardium becomes an anatomically well-demarcated two-layered epithelium and the pre-cardiac ECM becomes split into two compartments: (1) An intramyocardial ECM (marked by asterisks) and (2) a cell-free myoendocardial ECM (CJ).

The timing of the first appearance of an anatomically well-demarcated myoendocardial space depends on the structural development of the primary myocardium and the formation of endocardium-lined tubes (Figure 3). Both components of the wall of the embryonic heart tube derive from bilaterally paired areas of the visceral layer of the lateral plate mesoderm. These areas are called the “pre-cardiac mesoderms” or the “heart fields”. The pre-cardiac mesoderms harbor two different progenitor cell populations. These are (1) myocardial progenitors (pre-myocardium), which form the coelomic epithelium of the heart fields, and (2) endocardial progenitors (pre-endocardium), which are found in close spatial association with the endoderm. The pre-cardiac mesoderms, furthermore, harbor a gelatinous ECM, which connects the pre-myocardium and pre-endocardium with each other, and with the endoderm (Figure 3). This ECM layer, which likely becomes incorporated into the CJ of the prospective heart tube, was called CJ in a few previous publications, e.g., [37,38]. However, we prefer the usage of the term, “pre-cardiac ECM”. This is mainly for the following reason. The pre-myocardium does not possess a continuous basal border to the pre-cardiac ECM, and the pre-endocardium is a population of scattered mesenchymal cells embedded in the pre-cardiac ECM (Figure 3). Consequently, the pre-cardiac mesoderms initially do not possess well-formed anatomic borders that demarcate a myoendocardial space devoid of cells. The pre-cardiac ECM has the character of an intercellular substance that glues together the cellular elements of the pre-cardiac mesoderms and, therefore, should not be regarded as a cell-free ECM compartment. The appearance of an anatomically well-demarcated myoendocardial space depends on the differentiation of the pre-myocardium and pre-endocardium into the primary myocardium and endocardium-lined tubes, respectively (Figure 3). During myocardial differentiation, the initially discontinuous basal layer of the pre-myocardium becomes transformed into a continuous cell sheet bordered by a basal lamina. Consequently, the developing myocardium becomes an anatomically well-demarcated two-layered epithelium and the pre-cardiac ECM becomes split into two compartments: (1) A small compartment, which fills the intercellular spaces within the primary myocardium (intramyocardial ECM), and (2) a large, cell-free compartment, which fills the developing myoendocardial space (cardiac jelly).

It is well known that the chronology of the morphogenetic processes contributing to early cardiogenesis differs markedly across vertebrate species [40,41]. It is, therefore, no wonder that the relation between the timing of the first appearance of an anatomically well-demarcated myoendocardial space and the timing of the emergence of the embryonic heart shows species-specific differences. The initially straight heart tube of vertebrate embryos is a single median structure that arises from the union of materials from bilaterally paired heart fields in front of the developing foregut [42]. In some mammalian species, such as rabbits [43,44], rats [45,46], cats [47], and tree screws [41], myocardial differentiation and the establishment of the primary heart wall architecture precedes the union of the paired heart fields. In these species, a bilateral pair of endocardial tubes, surrounded by CJ and a contracting myocardial mantle, is formed within the two heart fields shortly before the latter start their union along the ventral midline of the foregut to form the single median heart tube. In this situation, the median heart tube seems to arise from the union of a pair of actively pulsating blood vessels, which have been named “lateral hearts” [43,44,45,46]. In other vertebrate species, such as chicks [48,49], the frog, *Xenopus laevis* [50], mice [38], and human beings [4], however, the establishment of the primary heart wall architecture occurs in the median heart tube, which means after the heart fields have started their union. In these species, the first contractions of the developing myocardium were recorded in the median heart tube [39,51,52,53]. Thus, comparative embryology shows that the establishment of an anatomically well-demarcated myoendocardial space, filled with CJ, obviously, does not depend on the establishment of a single median heart tube, but rather correlates with the structural and functional differentiation of the myocardium and endocardium.

**Figure 4:**
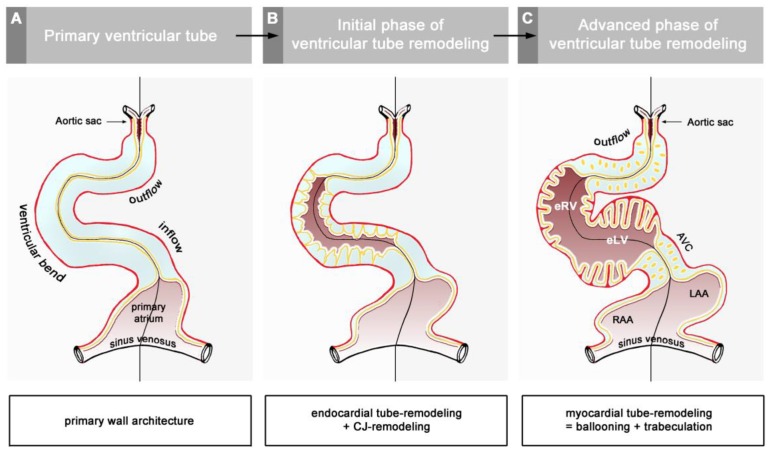
These schematic drawings illustrate three subsequent phases in the development of the wall architecture of the median embryonic heart tube of higher vertebrates. Note that, for reasons of simplification, this scheme neglects some developmental changes in the size and 3D-configuration of the heart (e.g., elongation of outflow, complex ventricular looping). (**A**) Primary wall architecture (red = primary myocardium; light blue = CJ; yellow = primary endocardium). Local variations in the thickness of the CJ layer facilitate the distinction of the two main building units: (1) A sac-shaped venous chamber, which has only a thin CJ layer and represents the primary atrium; and (2) a tube-shaped arterial conduit, which has a thick CJ layer and represents the primary ventricular tube. The presence of a thick CJ layer facilitates complete end-systolic occlusion of the endocardial lumen of all portions of the primary ventricular tube. The paired pattern of CJ accumulation reflects the origin of the ventricular tube from bilaterally paired heart fields. (**B**) The initial phase of the ventricular tube remodeling is characterized by remodeling of the endocardial tube and CJ along the convexity of the ventricular bend. Endocardial sprouts/protrusions invade the CJ and grow toward the primary myocardium. Consequently, CJ disappears along the convexity of the ventricular bend. (**C**) The advanced phase of the ventricular tube remodeling is characterized mainly by remodeling of the myocardium along the convexity of the ventricular bend and by remodeling of the CJ in the ventricular inflow (atrio-ventricular canal) and outflow portions. Endocardial protrusions invade the myocardial wall, which starts centrifugal growth (ballooning and trabeculation). The CJ in the atrio-ventricular canal (AVC) and outflow does not disappear, but becomes remodeled into endocardial cushions/ridges due to the invasion of endocardium-derived mesenchymal cells. Abbreviations: eLV = embryonic left ventricle; eRV = embryonic right ventricle; LAA = anlage of left atrial appendage; RAA = anlage of right atrial appendage.

The union of the bilaterally paired heart fields/lateral hearts starts at the level of the prospective apical trabeculated regions of the ventricles from where it progresses toward the venous as well as arterial poles of the single median heart tube, thereby adding the primordia of further building elements (atrio-ventricular canal, atriums, sinus venosus, ventricular outflow) to the developing heart [42]. During the initial steps of heart field union, no significant differences in the wall architecture are found along the length of the forming median heart tube. At advanced stages of the establishment of the median heart tube, however, regional differences in the thickness of the CJ layer become apparent along its length, which facilitate the distinction of the two main building units. These units are (1) a sac-shaped venous chamber, which has only a thin layer of CJ, and (2) a tube-shaped arterial conduit, which has a thick layer of CJ (Figure 1C and Figure 4A). The sac-shaped unit gives rise to the prospective atria and, therefore, may be called the “primary atrium”, while the tube-shaped conduit gives rise to the prospective ventricles and, therefore, may be called the “primary ventricular tube” [6,9,54,55]. Cardiac looping morphogenesis transforms the initially straight ventricular tube into a looped ventricular tube consisting of three portions: (1) Inflow, (2) ventricular bend, and (3) outflow. Due to the presence of a thick CJ layer, the inflow and outflow portions possess relatively well-defined anatomical borders to the primary atrium and aortic sac, respectively. They do not possess, however, well-defined anatomical borders to the ventricular bend.

**Figure 5:**
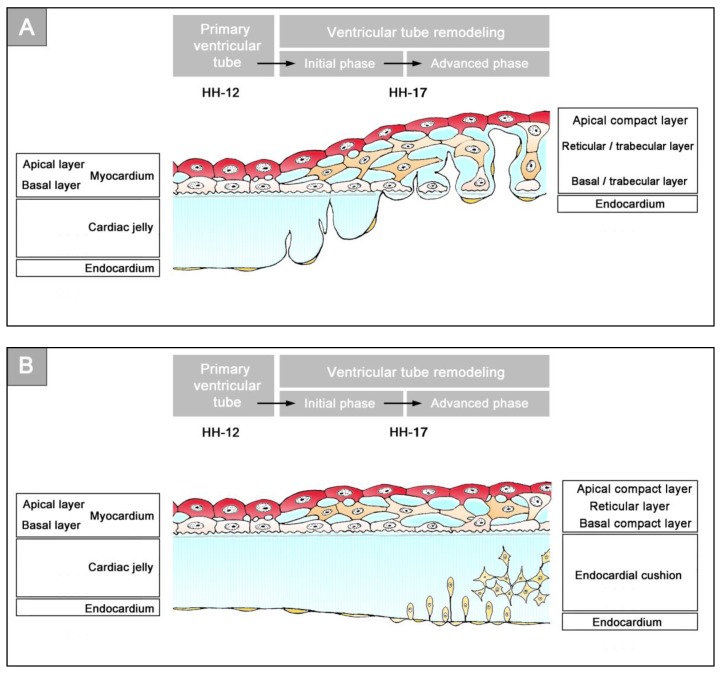
These schematic drawings illustrate the region-specific remodeling of the primary ventricular tube of higher vertebrate embryos as seen in the chick (based on data from Manasek [56] and Icardo and Fernandez-Terán [57]). (**A**) Remodeling along the outer curvature of the ventricular bend. CJ disappears due to the outgrowth of multiple endocardial pouches from the endocardial tube (initial phase of remodeling). When the endocardial pouches reach the basal layer of the myocardium, they start an invasion of the myocardial wall. Now, remodeling is no longer confined to the endocardium and CJ, but, additionally, changes the wall architecture of the myocardial tube. The initially smooth inner myocardial wall becomes transformed into a trabeculated myocardial wall (advanced phase of remodeling). (**B**) Remodeling in the inflow and outflow portions of the ventricular tube. CJ does not disappear, but loses its cell-free phenotype due to the invasion of endocardium-derived mesenchymal cells. Cellularized CJ is named endocardial cushions (in the inflow portion) or endocardial ridges (in the outflow portion).

The thick layer of CJ, characterizing the primary wall architecture of the ventricular tube, facilitates complete end-systolic occlusion of the endocardial lumen of all portions of the ventricular tube (inflow, ventricular bend, outflow) and, thereby, supports the initial pumping action of the valveless embryonic heart [16]. Subsequent to the establishment of a hemodynamically effective pulsatile blood flow, however, a remodeling process starts within the ventricular tube that transforms its primary wall architecture and finally leads to the disappearance of a cell-free, gelatinous ECM (CJ). This remodeling process proceeds from the inside to the outside of the ventricular tube and can be subdivided into two phases, which we call the “initial” and “advanced phase of ventricular tube remodeling” (Figure 4B,C). The initial phase of ventricular tube remodeling is characterized mainly by regionally confined remodeling of the endocardial tube and its surrounding CJ. Except for internal structural changes (formation of a reticular layer), the myocardial tube does not significantly change the primary smooth-walled phenotype of its apical and basal surfaces (Figure 4B and Figure 5A,B). The architectural changes of the endocardial tube and CJ are regionally confined to the convexity of the ventricular bend. Here, the endocardial tube forms multiple sprout- or pouch-shaped outgrowths that invade the CJ and grow toward the basal surface of the primary myocardium. Consequently, CJ disappears along the convexity of the ventricular bend [6,56,57,58]. The advanced phase of the ventricular tube remodeling is characterized mainly (1) by remodeling of the myocardium along the convexity of the ventricular bend and (2) by remodeling of the CJ in the inflow and outflow portions of the ventricular tube (Figure 4C and Figure 5A,B). The above-mentioned endocardial sprouts/pouches now invade the myocardial wall along the convexity of the ventricular bend and establish a primitive blood supply to the developing myocardium. The affected areas of the ventricular myocardial wall undergo an aneurysm-like expansion—nowadays called ventricular “ballooning” [59]—and acquire a trabeculated wall architecture [6,57]. The ventricular bend, thereby, loses its primary tubular shape and starts the formation of externally visible ventricular chambers. In contrast to the convexity of the ventricular bend, the inflow and outflow portions of the ventricular tube keep their primary tubular shape as well as their primary thick myoendocardial spaces for a relatively long time. The CJ of these regions, however, becomes invaded by endocardium-derived mesenchymal cells and, thereby, loses its cell-free phenotype and changes its biophysical properties (e.g., stiffness) [27,60,61]. The paired accumulations of cellularized CJ are usually named “endocardial cushions”. While some investigators used this term for the cellularized CJ of the inflow as well as the outflow portions others used this term only for the cellularized CJ of the inflow portion and called those of the outflow portion “endocardial ridges” [62,63]. In the following, we will use the latter terminology.

Having presented an overview on the chronology of CJ morphogenesis, we will now describe the morphological dynamics of the CJ and its biomechanical implications during the above-mentioned three phases of early heart wall development.

## 3. Morphology and Function of the CJ in the Primary Ventricular Tube

In the preceding paragraphs, we have seen that, in the so-called heart tube of vertebrate embryos, only the ventricular component has a tubular shape and a thick CJ layer while the atrial component is sac-shaped and has only a thin CJ layer. These differences in the shape and wall architecture are associated with differences in the contraction behavior of the two components. While the wall of the primary atrium contracts in a more or less synchronous fashion, the wall of the primary ventricular tube contracts in the fashion of a traveling wave that starts at the ventricular inflow and travels along the tube toward the end of its outflow portion. At the present time, it is unclear whether these traveling waves reflect a peristaltic- or Liebau effect-driven pumping action of the primary ventricular tube [1]. With respect to the biomechanical function of the ventricular CJ, however, this fact does not seem to be a problem, since analyses on heart tube models have shown that both pumping mechanisms profit from the presence of a thick layer of CJ [16,64].

**Figure 6:**
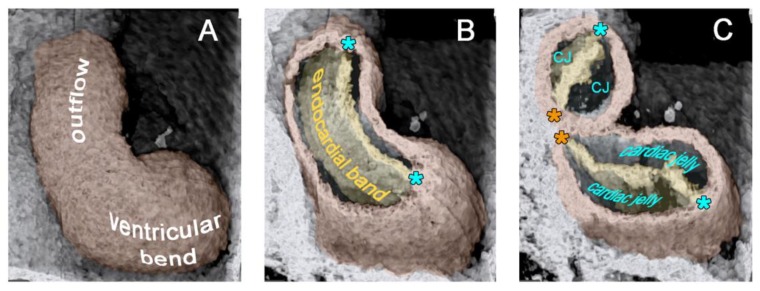
These 3D-images, acquired in vivo by 4D optical coherence tomography (OCT) [73], show various aspects of the wall architecture of the primary ventricular tube of a HH-stage 12/13 chick embryo heart as seen in right lateral views. Pictures show the ventricular bend and outflow in a fully contracted (end-systolic) state. (**A**) Outer shape of the myocardial tube. (**B**) 3-D shape of the occluded endocardial tube as shown by virtual removal of parts of the myocardial wall. Note that the collapsed endocardial tube has the shape of a flat band, whose edges are fixed to the original ventral and dorsal midlines of the heart tube (marked by blue and orange asterisks, respectively). (**C**) Cross-sections of the ventricular tube at the level of the ventricular bend and outflow as depicted by virtual dissection of OCT datasets. Note that the flattened endocardial tube is flanked by paired accumulations of CJ, which have half-moon shaped cross-sections. The paired character of the CJ-layer reflects the origin of the ventricular tube from the bilaterally paired heart fields.

Based on geometrical analyses, Barry has shown that the thick layer of CJ acts as a passive filler substance that facilitates complete end-systolic occlusion of the ventricular endocardial tube at physiological rates of myocardial shortening [16]. His calculations were based on hypothetical heart tubes of simplified geometry in which all three wall-layers had a perfect circular cross-section. This means that the CJ-layer was of a uniform thickness around the wall of the hypothetical heart tube (Figure 2B). Similar geometrical considerations were carried out by other researchers to demonstrate general principles in the functional architecture of the contracting wall of biological pumps and sphincters [65,66]. In contrast to the simplified hypothetical conduits used by Barry [16] and others [65,66], however, the primary wall of a real embryonic heart tube is not of a uniform thickness in the cross-section (Figure 1A,B). This is the consequence of a non-uniform spatial distribution of the CJ. The original ventral and dorsal midlines of the ventricular tube—the “fusion” lines of the paired heart fields—show only a very thin layer of CJ, while marked accumulations of CJ are found along the original left and right lateral walls of the ventricular tube [4,16,38,67,68,69,70,71,72]. Thus, the CJ of the ventricular tube shows a paired spatial patterning that reflects its origin from the bilaterally paired heart fields. The paired character of the CJ distribution is found during all phases of the cardiac cycle. It is particularly prominent, however, at the end-systolic occlusion of the endocardial tube when the CJ has the greatest thickness during the cardiac cycle (Figure 6).

**Figure 7:**
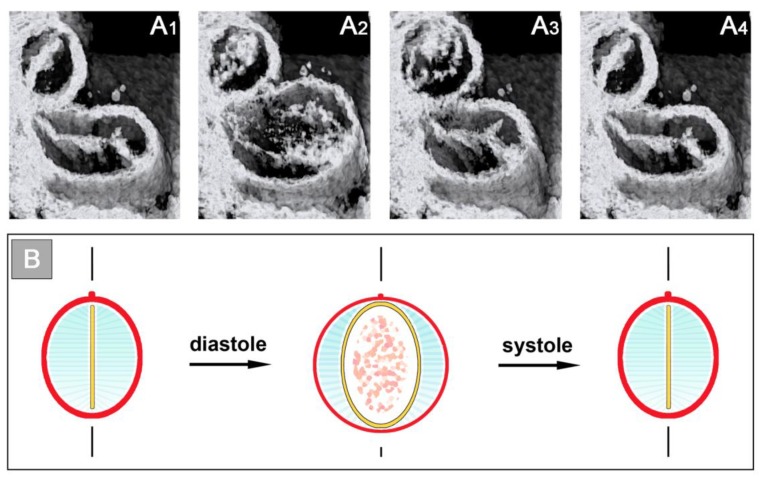
Cardiac cycle-related changes in the cross-sectional shape of the primary ventricular tube as visualized (**A**) by in vivo 4D OCT imaging of a HH-stage 12/13 chick embryo heart (see Figure 6C); and shown (**B**) by schematic drawings (same color code as used in Figure 2). Note that, due to the non-uniform spatial distribution of the CJ, the endocardial tube does not show concentric widening and narrowing during the cardiac cycle. The opened endocardial tube has an elliptic cross section, while the collapsed endocardial tube has a slit-shaped cross section. Note also the cyclic changes in the thickness of the CJ layer. The CJ undergoes thinning during relaxation of the surrounding myocardial wall (diastole) and thickening during contraction of the myocardial wall (systole).

The occluded endocardial tube has the shape of a flat band that marks the original mid-sagittal plane of the heart tube (Figure 6B). This band is flanked by paired accumulations of thickened CJ, which have half-moon shaped cross-sections. The endocardium and CJ are surrounded by the contracted myocardial wall, which has an elliptic cross-section (Figure 6C). During the filling phase of the ventricular tube (diastole), the collapsed lumen of the endocardial tube re-expands and acquires an elliptic cross-section flanked by a pair of sickle-shaped accumulations of CJ. The surrounding myocardial wall is in a relaxed state and acquires an almost circular cross-section (Figure 7).

In view of the fact that the non-uniform spatial distribution of CJ reflects the origin of the ventricular tube from bilaterally paired heart fields, it is tempting to suspect that the cross-sectional shape of the primary ventricular tube may represent no more than a functionally insignificant architectural relict of the preceding union of the paired heart fields. Analyses on heart tube models, however, have shown that, compared to a ventricular tube model with a circular wall architecture, a ventricular tube model with an elliptic lumen, due to the non-uniform CJ distribution, produces a 30% higher area reduction for the same amount of contraction energy introduced into the model system [74]. Thus, the physiologically non-uniform distribution of CJ around the primary ventricular wall can be explained, not only in terms of the union of the bilaterally paired heart fields, but, additionally, in terms of an improvement of the mechanical pumping efficiency. Besides the solid-dynamic advantages, the non-uniform architecture of the primary ventricular wall may also have fluid-dynamic advantages. Sarin and Mehrotra, for example, have shown that, under certain conditions, the resistance to the flow in a tube of an elliptic cross-section can be markedly lower than in a tube of a circular cross-section [75]. Future studies are needed to test this idea.

**Figure 8:**
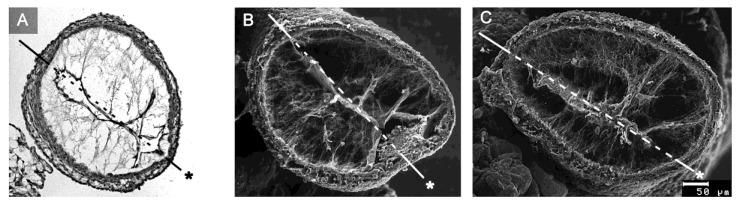
These images depict the architecture of the fibrillar components of the CJ as seen on cross-sections of the ventricular tube of embryonic chick hearts (HH-stage 16). (**A**) Routine histological section (HE staining) at the level of the ventricular bend; and (**B**,**C**) scanning electron microscopic pictures of cross-sections at the level of the ventricular bend (**B**) and ventricular inflow (**C**). Hearts were fixed at end-systole when the CJ has the greatest thickness during the cardiac cycle. Broken lines mark the closing planes of the collapsed endocardial tubes. Asterisks mark the original ventral midline of the ventricular tube. The CJ fibrils form a delicate network that is anchored to the basal laminae of the primary endocardium and myocardium. Within this network of interconnected fibrils, the principal fiber orientation is perpendicular to the basal surfaces of the endocardial and myocardial tubes. During reopening of the endocardial tube (diastole), the endocardial surfaces curve and the CJ fibrils acquire a principally radial orientation around the center axis of the ventricular tube (not shown).

The above-mentioned analyses on embryonic heart tube models have shown that the CJ is needed for proper systolic function of the primary ventricular tube. Based on in vivo observations on the heart tubes of chick embryos, Barry had suggested that the CJ might also support the diastolic filling of the primary ventricular tube [16]. He had observed that if a HH-stage 11/12 chick embryonic heart tube is transsected caudal to the venous heart pole, coelomic fluid is sucked into the opened heart during the refilling (diastole) phase of the still beating ventricular tube. The sucking motion of the primary ventricular tube may be the dominant filling mechanism during the initial phase of embryonic heart action, since, at HH-stage 12, less than 10% of the filling volume of the primary ventricular tube of a chick embryonic heart results from active contraction of the primary atrium [76]. Barry has speculated that the diastolic reopening of the lumen of the primary ventricular tube is powered by the release of passive energy stored in the elastic deformation of compressed CJ during the preceding systole [16]. Besides the statement that the CJ is a resilient substance, however, he did not present any idea about the structural basis for the suspected elastic restoration of the relaxed (diastolic) shape of non-compressed CJ. Thirty years later, however, Nakamura and Manasek attributed the diastolic reopening of the endocardial tube to formed components of the CJ, namely the so-called CJ fibrils [77]. At the time of Barry’s pioneering work, the existence of CJ fibrils, as normal histological structures, was questioned by some researchers. At that time, information about the internal architecture of the CJ was obtained only from the examination of routine histological sections of early embryonic heart tubes. In such preparations, fibrillated networks of a variable amount and texture are frequently (Figure 8A), but not regularly (Figure 1), found within the myoendocardial space.

While several investigators regarded these fibrillar networks as normal structural components of the CJ [3,11,55], other investigators interpreted such pictures as showing fixation artifacts, namely the coagula of a principally homogenous ground substance [4,9,27].

During the last three decades of the 20th century, several groups analyzed the biochemical nature and/or architecture of CJ fibrils by the use of modern microscopic techniques. The generally accepted conclusions arising from these studies are (1) that the fibrillar networks found in the myoendocardial spaces of fixed embryonic heart tubes are normal structural components of the CJ; (2) that the fiber system consists of collagenous as well as non-collagenous filaments; and (3) that the principal orientation of CJ fibrils is perpendicular to the basal surfaces of the primary endocardium and myocardium, which gives the impression of a predominantly radial orientation of the CJ fibrils in diastolic hearts [77,78,79].

With respect to the structural basis of the diastolic CJ function proposed by Barry, Nakamura and Manasek have speculated that, if the radial CJ fibrils are elastic, they should be stretched during systolic thickening of the CJ and thereby store a certain proportion of the myocardial contraction energy. When the myocardial wall relaxes, this energy should be released and drive the passive widening of the wall of the primary ventricular tube during diastole [77]. In support of this speculation, they found that radial CJ fibrils appeared to be wavy in relaxed (diastolic) hearts and stretched in contracted (systolic) hearts [77,78]. Besides the proposed role in the diastolic re-expansion of the primary ventricular tube, the CJ fiber system may have further biomechanical functions. Garita and co-workers, for example, have proposed that the system may act as a three-dimensional mechanosensing network used by the primary endocardium and myocardium [72]. This idea is in accord with the new conceptual hypothesis that the microfibrillar components of elastic ECMs act as structural tensometers that facilitate cells to sense and respond to changes in tissue mechanics [80].

The dynamic behavior of the CJ, described in the hypothetical scenario of Nakamura and Manasek, show striking similarities to the behavior of the ECM of jellyfish during swimming motions. Jellyfish is the informal name given to the medusa-stage of certain soft-bodied marine invertebrates. Jellyfish are mostly free-swimming animals that have a bell- or umbrella-shaped body form. Similar to the situation in the primary ventricular tube of vertebrate embryonic hearts, the soft body of jellyfish is mechanically stabilized by a hydraulic skeleton, made up of a transparent, highly hydrated form of gelatinous ECM. This viscoelastic ECM is called “mesoglea” [23,81]. The mesoglea is interposed between the outer (epidermis) and inner (gastrodermis) skins of the umbrella-shaped body. Corresponding to the CJ, the mesoglea harbors a system of vertically/radially oriented ECM fibers that consists of collagenous as well as non-collagenous components [81,82,83]. Non-collagenous ECM fibers contribute to the elastic properties of the mesoglea and have been identified as fibrillin-rich fibers [83,84]. Jellyfish swim by radial contractions and expansions of their umbrella-shaped bodies. Contraction of the body narrows the lumen of the subumbrellar cavity so that a quantity of its water content is ejected rearwards through the opening of the cavity and the animal is propelled forward. During the subsequent expansion of the body, the subumbrellar cavity widens so that water is sucked back into the subumbrellar cavity. Contraction of the jellyfish body is an active process generated by the action of a circular swimming muscle. It is accompanied by a significant increase in the thickness of the mesoglea. Expansion, on the other hand, is a passive process driven by the release of energy stored in the elastic deformation of the mesoglea during the preceding contraction. Expansion of the jellyfish body is accompanied by a thinning of its mesoglea layer. The elastic restoration of the resting shape of the jellyfish body is mainly attributed to the radially oriented fibers of the mesoglea. These fibers become stretched during contraction-induced thickening of the mesoglea and re-shorten during expansion of the jellyfish body [83,85].

The above-mentioned observations on jellyfish suggest that the CJ has a functional design that is widely used in the animal kingdom. We should note, however, that some experimental results seem to cast doubt on the postulated diastolic function of the CJ. Baldwin and co-workers [86] have analyzed the effects of CJ removal (by hyaluronidase treatment) on the ventricular function of the post-looped heart tube of rat embryos. They have found that, compared to normal controls, CJ-deficient heart tubes show a significantly faster diastolic refilling of the ventricular bend. They have concluded that this finding seems to disprove Barry’s concept of a passive, CJ-driven refilling of the primary ventricular tube. We should note here, however, that the higher velocity of diastolic refilling, observed in CJ-deficient heart tubes, can be simply explained by the backflow of blood from the aortic sac due to the failure of CJ-dependent occlusion of the ventricular outflow portion. The outflow portion normally is in a contracted (systolic) state during refilling (diastole) of the ventricular bend and, thereby, prevents the backflow of blood from the aortic sac. We should furthermore note that the elastic restoration of the relaxed shape of the heart tube, which is proposed to drive diastolic refilling of the heart, can be observed in the beating heart tubes of chick embryos whose cardiovascular systems have been emptied of blood by the opening of their vitelline vessels [58].

## 4. Morphology and Function of the CJ during the Initial Phase of Ventricular Tube Remodeling

The primary wall architecture (smooth-walled myocardial tube, CJ layer of a non-uniform thickness, smooth-walled endocardial tube of the elliptic diastolic cross-section) is found along the entire length of the ventricular tube only during a relatively short phase of early cardiac pumping action. In chick embryos, this phase spans the developmental period from HH-stage 10/11 to HH-stage 12/13, and in human embryos, it spans the developmental period defined by Carnegie stage 10. Subsequent to the establishment of a hemodynamically effective pulsatile blood flow, a remodeling process starts within the ventricular tube that transforms its primary wall architecture. During the initial phase of ventricular tube remodeling, changes in the wall architecture are confined to the endocardial tube and CJ along the outer curvature (convexity) of the ventricular bend. The wall of the inner curvature (concavity) of the ventricular bend as well as the walls of the ventricular inflow and outflow portions do not show significant changes of their primary architecture. This remodeling pattern corresponds to the spatial distribution of high (inflow, inner curvature, outflow) and low (outer curvature) shear stress areas found within the endocardium of the looped ventricular tube of chick embryos (see Figure 1 in [87]), suggesting that hemodynamic forces may trigger the remodeling process.

Remodeling starts with the formation of multiple sprout- or pouch-like outgrowths of the endocardial tube that invade the CJ and grow toward the basal layer of the myocardium [4,6,56,57,58]. Scanning electron microscopic data from chick embryos disclosed an intimate physical connection between the endocardial sprouts and the radially oriented CJ fibers, suggesting that the latter might be used as guidance by the former [58].

Concomitant with the outgrowth of endocardial sprouts, the thickness of the CJ layer is reduced along the outer curvature of the ventricular bend and the tips of the endocardial sprouts eventually come into direct contact with the basal surface of the myocardium. These endocardial-myocardial contacts are established exactly in those areas where the myocardial wall will expand and develop a trabeculated architecture during the subsequent phase of advanced ventricular tube remodeling. Davis and Streeter, therefore, called the endocardial outgrowths the “primordia of endothelial trabeculae” [4,6]. In chick embryos, the initial phase of ventricular tube remodeling spans the developmental period from HH-stage 13 to HH-stage 16/17, and in human embryos, it spans the developmental period defined by Carnegie stage 11.

The invasion of multiple endocardial protrusions into the CJ leads to the establishment of two broad areas of diminished CJ along the outer curvature of the ventricular bend, which mark the positions of the prospective apical trabeculated regions of the left and right ventricles. In the following, these areas will be termed “trabecular shields”. The comparison of data from human and chick embryos discloses species-specific differences in the chronology of the establishment of the trabecular shields. In human embryos, the formation of trabecular shields starts at the level of the prospective left ventricle [6], while in chick embryos, this process starts at the level of the prospective right ventricle [88,89]. These differences may reflect species-specific differences in the chronology of the union of the bilateral heart fields. In mammalian embryos, the union of the heart fields starts at the level of the prospective apical trabeculated regions of the left ventricle [42], while in chick embryos, this process is said to start at the level of the prospective apical trabeculated region of the right ventricle [90].

**Figure 9:**
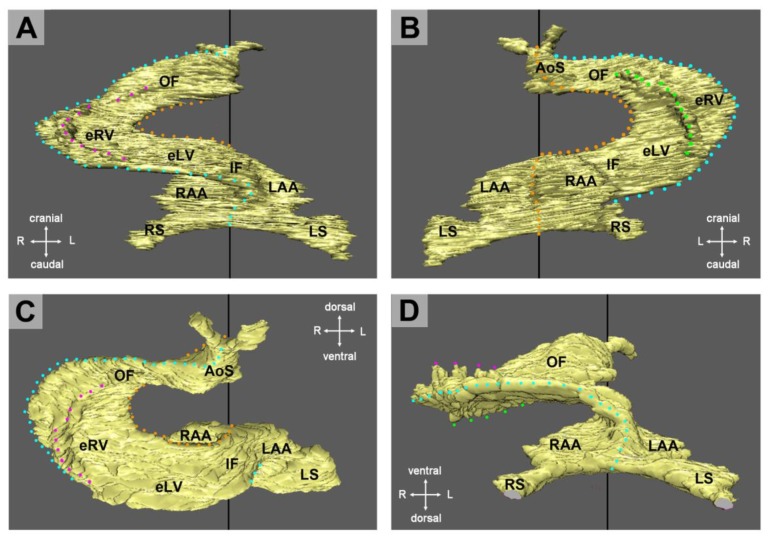
These pictures show the outer shape of an occluded endocardial tube of an embryonic chick heart during the initial phase of ventricular tube remodeling (HH-stage 14). Images were generated by virtual 3-D reconstructions of OCT data sets from a heart that has been fixed in an artificially induced general contraction. (**A**) Frontal view. (**B**) Dorsal view. (**C**) Cranial view. (**D**) Caudal view. The outer curvature of the ventricular bend has a folded surface, which is characterized by three folds: (1) A midline fold that corresponds to the original ventral edge of the occluded endocardial tube (marked by lines of blue dots). This fold subdivides the trabecular shields into two halves. (2, 3) A pair of lateral folds that form the lateral borders of the trabecular shields (marked by dotted lines in magenta and green). Abbreviations: eLV = embryonic left ventricle; eRV = embryonic right ventricle; IF = inflow portion of ventricular tube; RAA = anlage of right atrial appendage; RS = right horn of sinus venosus; LS = left horn of sinus venosus; OF = outflow portion of ventricular tube.

**Figure 10:**
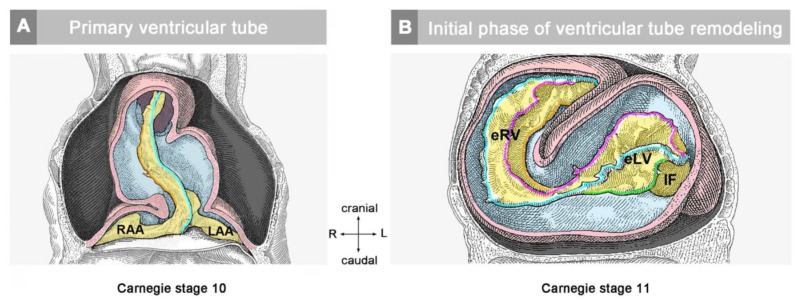
These drawings depict the changes in the outer shape of the occluded (systolic) endocardial tube of human embryonic heart tubes during the initial phase of ventricular tube remodeling. Hearts are shown in frontal views within the opened pericardial cavities. The ventral myocardial walls have been removed to facilitate views on the endocardial tubes. (**A**) The occluded endocardial tube of the primary ventricular tube has the shape of a flat band. Its ventral (blue line) and dorsal (orange line) edges correspond to the original ventral and dorsal midlines of the heart. (**B**) Due to the formation of trabecular shields, the occluded endocardial tube has a folded surface along its outer curvature. Corresponding to the situation in chick embryonic hearts (Figure 9), the picture is dominated by the presence of three folds: (1) A midline fold that corresponds to the original ventral edge of the occluded endocardial tube (blue line). This fold subdivides the trabecular shields into two halves. (2 + 3) A pair of lateral folds that form the lateral borders of the trabecular shields (magenta and green lines). Drawings are based on Figures 22 and 26 from Davis [4]. Same abbreviations as used in Figure 9.

Historical descriptions do not mention any internal patterning of the trabecular shields. However, our studies on chick embryos have shown that these areas have a regular spatial patterning that is best visualized by three-dimensional imaging of the outer shape of the collapsed (systolic) endocardial tube (Figure 9). This pattern is also found on historical pictures from human embryonic heart tubes (Figure 10), but, for unknown reasons, has escaped the attention of previous investigators.

As already described, the collapsed endocardial tube of the primary ventricular tube has the shape of a flat band whose edges correspond to the original ventral and dorsal midlines of the heart tube (Figure 6B and Figure 10A). Due to the formation of the trabecular shields, the outer shape of the collapsed endocardial tube of the ventricular bend no longer appears as a flat band, but shows a folded surface along its outer curvature (Figure 9 and Figure 10B). The picture is dominated by the presence of three regularly formed folds. The first of these folds corresponds to the original ventral (midline) edge of the flattened primary endocardial tube. It subdivides the trabecular shields into two halves, which occupy the outer curvatures of the original left and right halves of the ventricular tube, respectively. The remaining two folds form the lateral borders of the trabecular shields. Based on this finding, it becomes apparent that each trabecular shield is a bilaterally paired structure.

**Figure 11:**
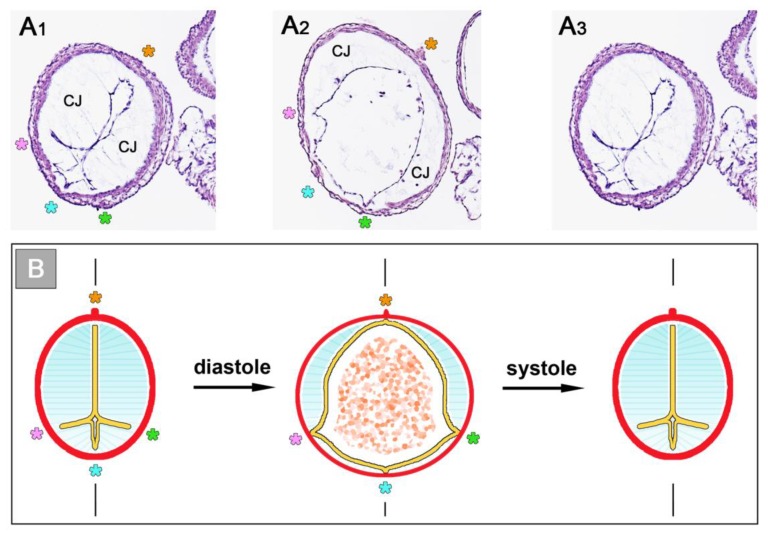
Initial phase of ventricular tube remodeling. Cardiac cycle-related changes in the cross-sectional shape of the ventricular bend as illustrated (**A**) by histological sections of HH-stage 16 chick embryonic hearts, which have been fixed in emptied (**A_1_,A_3_**) and filled (A_2_) states; and shown (**B**) by schematic drawings (same color code as used in Figure 2). Asterisks mark the original ventral (light blue) and dorsal (orange) midlines of the heart as well as the lateral borders of the trabecular shields (magenta and green). Note that, due to the formation of the trabecular shield, the opened endocardial tube has a bell-shaped or deltoid cross section, while the cross section of the collapsed endocardial tube has the shape of a Latin cross. Note also the cyclic changes in the thickness of the CJ layer.

The functional significance of early ventricular wall remodeling has not been clarified up to now. It is evident that the formation of the trabecular shields leads to an increase of the end-diastolic volume of the endocardial tube of the ventricular bend. This might support the pumping function. An argument against this idea may be the fact that the increase in the end-diastolic volume is at the expense of the CJ layer. Since a thick layer of CJ is needed for complete end-systolic occlusion of the endocardial tube, it is tempting to speculate that the formation of the trabecular shields might prevent complete end-systolic occlusion of the endocardial lumen and thereby reduces the original 100% ejection fraction. This might mitigate the positive effects of early ventricular wall remodeling. However, in vivo OCT imaging of the cardiac cycle of embryonic chick hearts has shown that, due to the above-mentioned system of endocardial folds, the endocardial lumen of the ventricular bend still undergoes complete end-systolic occlusion [58]. The cross section of the collapsed endocardial tube is no longer slit-shaped, but has the shape of a Latin cross. The descending arm of this cross corresponds to the closure plane of the primary endocardial tube while the horizontal arms represent the closure planes of the lateral folds. During diastolic refilling of the ventricular bend, the endocardial tube acquires a bell-shaped or deltoid cross section (Figure 11).

The above-mentioned studies of Taber and Perrucchio [74] have shown that the mechanical pumping efficiency of the early embryonic heart tube depends on the cross-sectional shape of its endocardial tube. Compared to a tube of a circular inner cross section, the natural design of a ventricular tube of an elliptic inner cross section was found to work at a higher mechanical efficiency. Early ventricular tube remodeling transforms the initially elliptic cross section of the opened endocardial tube of the ventricular bend into a deltoid cross section. At the present time, we do not know whether this transformation has any relevance for the pumping efficiency of the ventricular bend. We should note, however, that this shape change is accompanied by a general increase in the pumping performance of the embryonic heart tube [91]. Future studies are needed to clarify the relationships between the deltoid cross section and the pumping efficiency and hemodynamics of the ventricular bend.

**Figure 12:**
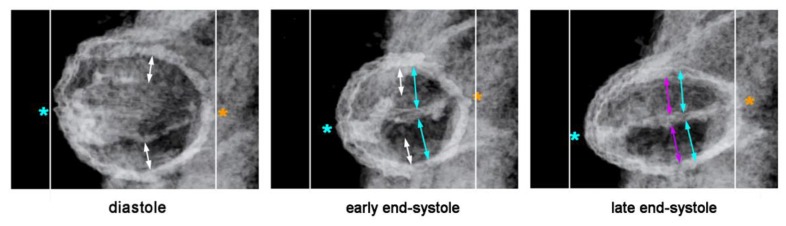
These images obtained by 4-dimensional in vivo OCT imaging of a HH-stage 15 embryonic chick heart depict the peculiar phenomenon of end-systolic stretching of the cross section of the ventricular bend along the original dorso-ventral axis. Asterisks mark the original ventral (light blue) and dorsal (orange) midlines, which are found along the outer and inner curvatures of the ventricular bend, respectively. Note the striking increase in the thickness of the CJ layer between diastole and early end-systole (indicated by white and blue arrows). Late end-systolic stretching of the cross section of the ventricular bend leads to a slight decrease in the CJ thickness (compare blue and magenta arrows). White lines mark the diastolic length of the original dorso-ventral axis.

Our reflections on the functional significance of the morphological dynamics of the ventricular bend would not be complete without referring to a peculiar phenomenon, which we have called “end-systolic stretching” [58]. Our data from in vivo imaging of the pump action of the ventricular bend of embryonic chick hearts have shown that the systolic deformations of the shape of the ventricular tube do not stop with the contact of opposing luminal surfaces of the emptied endocardial tube. Subsequent to end-systolic occlusion of the endocardial lumen, the ventricular wall becomes stretched along its original dorso-ventral axis, so that its outer cross-sectional shape becomes transformed from a circle into an oval (Figure 12). It is evident that this shape change is driven by end-systolic deformation of the CJ.

The phenomenon of end-systolic stretching, therefore, confirms Barry’s statement that the myocardial contraction energy is used not only for the propulsion of blood, but, additionally, for the elastic deformation of the CJ layer. End-systolic stretching is most prominent at the level of the ventricular bend and during the initial phase of ventricular tube remodeling [58]. This observation not only suggests that the ventricular bend generates the strongest contraction force of all the portions of the ventricular tube, but, additionally, may generate the highest proportion of the proposed sucking action of the ventricular tube.

## 5. Morphology and Function of the CJ during the Advanced Phase of Ventricular Tube Remodeling (Trabeculation, Ballooning, Endocardial Cushions/Ridges)

The time point of transition from the initial to the advanced phase of ventricular tube remodeling is reached when the endocardial projections of the trabecular shields have established direct contacts to the basal myocardial surface of the outer curvature of the ventricular bend. The capillary-like endocardial projections now penetrate the compact basal layer of the myocardium, invade the intercellular spaces of the loosely arranged middle (reticular) layer of the myocardial wall (Figure 5A), and start the establishment of a primitive blood supply to the developing myocardium [92]. The affected areas of the myocardial wall thereby lose their originally smooth basal surface and start the formation of a sponge-like inner layer whose muscular network is in intimate contact with branches of the endocardial projections. The sponge-like myocardium consists of myocardial trabeculations and intertrabecular spaces [56,57,92]. The intertrabecular spaces are occupied by the capillary-like endocardial projections, which traditionally have been named “endocardial trabeculations” [4,6,93] or “myocardial sinusoids” [94,95].

The formation of the trabecular layer of the ventricular myocardium is accompanied by centrifugal growth of the affected areas, which leads to the formation of aneurym- or balloon-like expansions of the ventricular tube along its outer curvature [59,93]. The ballooning portions of the ventricular bend are the first externally visible manifestations of the prospective left and right ventricle, and, therefore, are named the embryonic left and right ventricle, respectively. In chick embryos, the formation of myocardial trabeculae starts at HH-stage 17 [57,88,96], and in human embryos, it starts at Carnegie stage 12 [40,93].

The embryonic ventricles have lost the originally thick CJ layer and the original tubular shape of the ventricular bend. This is in contrast to the inflow and outflow portions, which still preserve the original tubular shape and still possess a thick myoendocardial tissue layer. Consequently, the inflow and outflow portions now have visible anatomical borders at their upstream and downstream ends. They have become anatomically well-demarcated units, which are named the “atrio-ventricular canal” (AVC) and “outflow tract” (OFT), respectively. The CJ of these tube-shaped units becomes remodeled into the so-called endocardial cushions/endocardial ridges by the invasion of endocardium-derived mesenchymal cells [27,60,61].

The above-mentioned changes in the anatomy of the developing heart are accompanied by changes in the pumping behavior of the primary ventricular tube derivatives. The balloon-shaped embryonic ventricles become the main pumping units of the developing heart and develop the mature patterns of electrical activation and contraction [97,98]. The AVC and OFT, on the other hand, act as tubular conduits, which (1) connect the embryonic ventricles with the developing atria and aortic sac, respectively; and (2) control intracardiac blood flow by the opening and closing of their lumen. The latter function of the AVC and OFT is based on a sphincter-like action and has the same effects on blood flow as the mature heart valves.

During the past decades, several excellent review articles have dealt with the above-mentioned processes of ventricular trabeculation/ballooning [99,100,101] and endocardial cushions/ridges development [102,103,104]. We, therefore, do not want to add a further extensive review about these processes in the present article. However, we think that there exist a few facts that are worth mentioning if we want to understand the relation between the CJ and the formation of endocardial cushions/ridges and myocardial trabeculations.

### 5.1. Notes on the Morphology and Function of Endocardial Cushions/Ridges

With respect to the relation between the CJ and endocardial cushions/ridges, we want to highlight the following facts: (1) Endocardial cushions/ridges arise from the remodeling of the non-removed CJ of the inflow and outflow portions of the primary ventricular tube [27]. They do not represent de novo formations. (2) The paired occurrence of endocardial cushions/ridges is based on the non-uniform spatial distribution of the CJ in the primary ventricular tube and, therefore, reflects the bilaterally paired origin of the whole heart. Thus, endocardial cushions/ridges are paired structures whose partners can be assigned to the original left and right halves of the straight ventricular tube, respectively [67,105,106,107]. (3) Endocardial cushions/ridges principally fulfill the same function as the CJ [27]. They act as passive filler tissues that facilitate complete occlusion of the endocardial lumen of the AVC and OFT during contraction of the myocardial wall. Their architecture and biomechanical properties are adapted to the increasing hemodynamic loads of the developing heart at late looping- and early post-looping stages [61]. (4) It is frequently stated that the CJ and endocardial cushions/ridges control blood flow by a valve-like action, e.g., [27,108]. We think that such a description may hamper the understanding of the pumping function of the valveless embryonic heart since it does not correctly reflect reality. The mature heart valves are biological devices that open and close the cardiac flow pathways in a purely passive manner driven by differences in blood pressure along the flow pathways. The basis for this behavior is the form design of heart valves, which normally permits one-way flow, only. This behavior is in sharp contrast to the action of the endocardial cushions/ridges. These biological devices do not open and close the endocardial lumen in a passive, blood pressure-driven way. Closure of the endocardial lumen of the AVC and OFT is an active process that is driven by contraction of the surrounding myocardial wall. During contraction of the myocardial wall, the endocardial cushions/ridges act as passive filler tissues facilitating complete occlusion of the endocardial lumen. Reopening of the lumen depends on relaxation of the myocardial wall. Furthermore, the form design of endocardial cushions does not determine the flow direction in the valveless embryonic heart. Instead, the flow direction is determined by the sequential contractions of the developing atrial and ventricular heart chambers. If the normal pattern of heart beat propagation is reversed, the flow direction is also reversed [109]. Such a behavior, as well as the wall architecture of the AVC and OFT, corresponds to biological sphincters [66]. We, therefore, think that the correct functional description of the action of the AVC and OFT is that of a sphincter-like action. This has been done previously by some investigators [63,99,110,111,112,113], but, for unknown reasons, did not receive general acceptance.

### 5.2. Notes on Ventricular Trabeculation and Ballooning

With respect to the relation between the CJ and the formation of ventricular trabeculations, we want to comment on two aspects. The first is the mechanism of the formation of myocardial trabeculations and the second is the ballooning model. 

**Figure 13:**
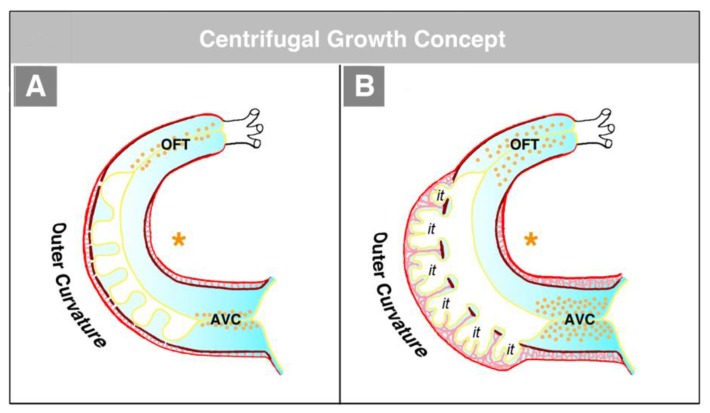
These schematic drawings illustrate the centrifugal growth concept of ventricular trabeculation. (**A**) Initial step of trabeculation. (**B**) Advanced step of trabeculation. According to this concept, myocardial trabeculae are formed mainly from cardiomyocytes of the middle (reticular) layer of the ventricular myocardium (marked by light red color). The endocardium-lined intertrabecular spaces (*it*) arise from the sprout-like endocardial protrusions of the trabecular shields. These sprouts penetrate the inner (basal) layer of the ventricular myocardium and start an invasion of the intramyocardial ECM of the reticular layer myocardium (**A**). Signals from the endocardium of the intertrabecular spaces stimulate growth of the reticular/trabecular myocardium, which leads to expansion (ballooning) of the embryonic ventricles (**B**). Note that the free edges of the myocardial trabeculae are remnants of the original inner (basal) layer of the primary myocardial tube. Color code: orange asterisks mark the inner curvature of the ventricular bend; dark red = remnants of the inner (basal) layer of primary myocardium; red = derivatives of the outer (apical) layer of the primary myocardium; light red = reticular layer of primary myocardium; light blue = CJ and intramyocardial ECM; yellow = endocardium. Abbreviations: AVC = atrio-ventricular canal; *it* = intertrabecular spaces; OFT = outflow tract.

The hearts of CJ-deficient mouse embryos do not form a trabeculated wall architecture, suggesting that the process of ventricular trabeculation depends on the presence of CJ [114]. The functional interpretation of this finding is made difficult by the fact that in the literature, two opposing concepts exist about the morphogenetic mechanism driving the formation of the trabeculated layer of the ventricular myocardium of vertebrate embryonic hearts. The first of these concepts corresponds to the description of the trabeculation process given in the preceding paragraphs of our present review article (Figure 4C and Figure 5A). It describes the formation and expansion of the trabecular layer of the embryonic ventricles as a centrifugal growth process [6,56,57,59,88,93,110,115]. This means that the myocardial trabeculae and the sprout-like endocardial projections principally elongate by outward growth toward the pericardial surface of the ventricular wall. The innermost portions of the trabeculated myocardium—the free edges of myocardial trabeculae—are regarded as remnants of the original basal layer of the primary myocardium, which do not significantly change their original positional relationship to the tube-shaped remainders of the primary ventricular tube (AVC and OFT) (see Figure 13). 

**Figure 14:**
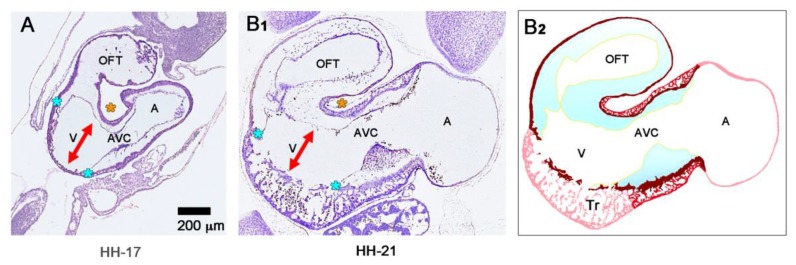
These histological sections from embryonic chick hearts depict the changes in the ventricular wall architecture during the advanced phase of ventricular tube remodeling. (**A**) At the time point of the transition from the initial to the advanced phase of ventricular tube remodeling (HH-stage 16/17), the outer curvature of the ventricular bend lacks a CJ layer and the tips of the endocardial projections of the trabecular shields are in direct contact to the basal layer of the ventricular myocardium (blue asterisks mark the proximal and distal borders of the trabecular shields; orange asterisk marks the inner curvature of the ventricular bend). In the AVC, endocardium-derived mesenchyme has started an invasion of the CJ. Note that the myocardial walls of all portions of the primary ventricular tube have a tubular shape. (**B_1_,B_2_**) HH-stage 21. Due to the formation of trabeculated embryonic ventricles, the outer contour of the ventricular bend has lost its original tubular shape. The AVC and OFT have preserved their original tubular shapes. When looking on the inner structure of the embryonic ventricles, however, it becomes apparent that the original tubular shape of the ventricular bend is still visible if one follows an imaginary line that connects the free edges of the myocardial trabeculae (highlighted by the dark red color in (**B_2_**). Note that the free lumen of the embryonic ventricles does not change significantly between HH-stages 17 and 21. Note also the structural similarity between the non-trabeculated, three-layered myocardium of the AVC (inner compact layer, reticular layer, outer compact layer) and the trabeculated myocardium of the embryonic ventricles. Color code used in (**B_2_**): dark red = remnants of the inner layer of the primary ventricular myocardium; red = reticular and outer compact myocardium of the AVC; light red = trabeculated and outer compact myocardium of embryonic ventricles, and atrial myocardium; light blue = CJ and intramyocardial ECM; yellow = endocardium. Abbreviations: A = primary atrium; AVC = atrio-ventricular canal; OFT = outflow tract; Tr = trabecular layer of ventricular myocardium; V = free lumen of the primary ventricular tube.

The centrifugal growth concept is founded on morphological data, which show that the free edges of the myocardial trabeculae form an imaginary line that follows the original course of the myocardial wall of the outer curvature of the primary ventricular tube (Figure 14) [6,88,93,110,115]. It is furthermore supported by experimental and molecular data suggesting that the edges of the myocardial trabeculae harbor the ontogenetically oldest cells directly derived from the myocardium of the primary ventricular tube [99].

**Figure 15:**
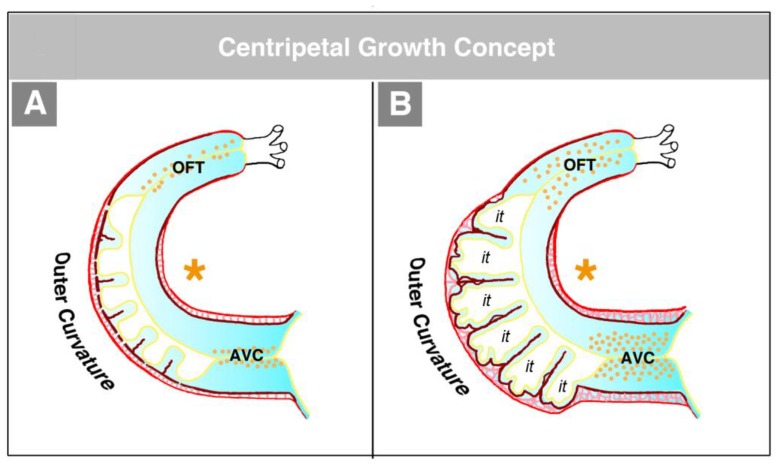
These schematic drawings illustrate the centripetal growth concept of ventricular trabeculation. (**A**) Initial step of trabeculation. (**B**) Advanced step of trabeculation. According to this concept, myocardial trabeculae are formed mainly from cardiomyocytes of the inner (basal) layer of the ventricular myocardium (marked by dark red color). Recent data suggest that non-removed remnants of CJ along the outer curvature of the ventricular bend serve as pathways for inward growth of the trabeculae toward the center of the embryonic ventricle. Inward growth of myocardial trabeculae separates endocardium-lined intertrabecular spaces (*it*) from the free ventricular lumen. Outward growth of endocardial sprouting into the myocardial wall does not significantly contribute to the formation of intertrabecular spaces. Note that inward growth of trabeculae is expected to narrow the free lumen of the embryonic ventricles during the initial step of trabeculation. Note also that the embryonic ventricles expand (ballooning) concomitant with the growth of the trabeculae. Same color code and abbreviations as used in Figure 13.

The second concept of the formation of myocardial trabeculae describes the emergence and expansion of the trabecular layer of the embryonic ventricles as a centripetal growth process [100,113,116,117,118]. This means that the myocardial trabeculae are suspected to elongate by inward growth toward the centers of the embryonic ventricles (see Figure 3 in [100], Figure 3 in [116], and Figure 1 in [117]). This inward growth is suspected to result either from a buckling process [100] or from the translocation of ventricular cardiomyocytes (by directional cell migration and oriented cell divisions) into non-removed CJ between the endocardial projections of the trabecular shields reviewed by [118]. The latter mechanistic concept suggests that the CJ might act as a physical route for the ingrowth of myocardial trabeculae. This idea could nicely explain the above-mentioned observation that CJ-deficient hearts do not form myocardial trabeculae [114]. We should note, however, that the inward growth concept conflicts with the morphological situation found in higher vertebrate embryos. If myocardial trabeculae would preferentially elongate by inward growth toward the centers of the embryonic ventricles, we should expect that the free lumen of the embryonic ventricles become continuously narrowed by the growing trabeculae (see schematic drawing in [117] and our Figure 15). As shown in our Figure 14, however, the diameter of the free ventricular lumen does not change significantly during the emergence of ventricular trabeculations.

In view of the arguments outlined above, it becomes apparent that we favor the centrifugal growth concept of myocardial trabeculations. With respect to the phenotype of CJ-deficient embryonic hearts, we should note that such hearts not only lack the CJ layer. They additionally show the abnormal phenotype of a multilayered compact myocardium that lacks any trace of reticular myocardium (see Figure 4 in [114]). Prior to the emergence of myocardial trabeculae, the ventricular myocardium normally thickens by the formation of an ECM-rich reticular layer, which is sandwiched between its outer (apical) and inner (basal) compact layers (Figure 5A). The absence of reticular myocardium shows that CJ-deficient hearts not only lack the myoendocardial ECM compartment (= CJ). They additionally lack the frequently neglected second ECM compartment of early embryonic hearts, namely the intramyocardial ECM. In view of this finding, the question arises as to whether the absence of myocardial trabeculae, found in cases of CJ-deficient hearts, might be explained by the absence of intramyocardial ECM rather than the absence of CJ. It is evident that the establishment of a reticular arrangement of cardiomyocytes depends on the production of intramyocardial ECM. We speculate that the production of intramyocardial ECM not only facilitates the formation of the reticular layer myocardium, but, additionally, facilitates the invasion of the endocardial projections into the ventricular myocardial wall, which eventually transforms the reticular myocardium into the so-called trabeculated myocardium.

**Figure 16:**
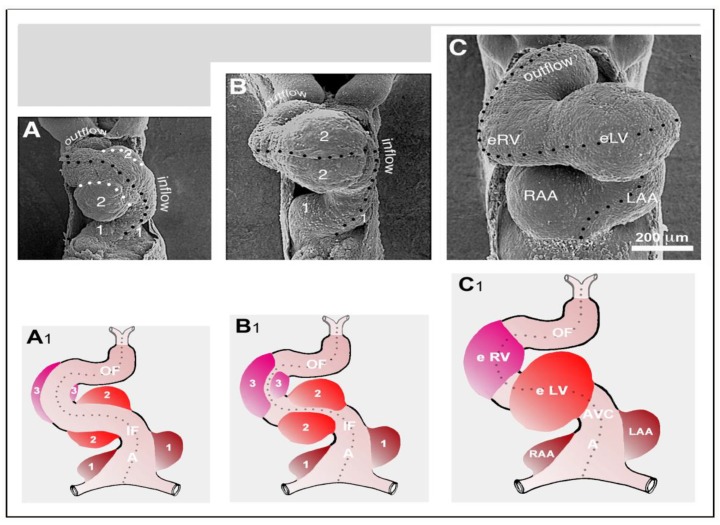
The modified model of cardiac chamber ballooning as depicted by changes in the outer shape of early embryonic hearts. (**A**–**C**) Scanning electron micrographs of embryonic mouse hearts on ED 8.5 (**A**), ED 9.0 (**B**), and ED 9.75 (**C**). Hearts are shown in fronto-caudal views. This facilitates direct imaging of the surface of the outer curvature of the ventricular bend. The black dotted lines mark the original ventral midline of the heart. Note that the inner curvature is not visible. (**A_1_**–**C_1_**) Schematic illustrations of the situations shown in Figures **A**–**C**. (**A,A_1_**) The initial steps of externally visible chamber ballooning are characterized by the emergence of bilaterally paired ballooning centers at three levels: (1) The embryonic atrium, (2) the prospective left ventricle, and (3) the prospective right ventricle. The white dotted lines in Figure A mark the ventral borders between the remnant of the primary ventricular tube and the paired ballooning centers of the prospective left ventricle. (**B,B_1_**) Due to expansion of the paired ballooning centers of the prospective ventricles, their ventral borders shift toward the original ventral midline of the ventricular bend. (**C,C_1_**) The originally paired ballooning centers of the prospective ventricles unite to form single units (embryonic left and right ventricle) that give rise to the apical trabeculated regions of the prospective ventricles. The paired ballooning centers of the embryonic atrium remain separated and give rise to the atrial appendages of the mature heart. Same abbreviations as used in Figure 9.

We finally want to comment on the relation between CJ morphogenesis and the ballooning model for cardiac chamber formation, which was introduced by the Amsterdam group in 2000 [59]. The ballooning model distinguishes four solitary ballooning centers that form along the outer curvatures of the primary atrium and primary ventricular tube. These centers are: (1) A right- and (2) a left-sided atrial center, which give rise to the atrial appendages of the prospective right and left atrium, respectively; and (3) a proximal and (4) a distal ventricular center, which give rise to the apical trabeculated regions of the prospective left and right ventricle, respectively. We principally agree with this elegant model, which is based on profound morphological as well as molecular data. Based on our present knowledge about the morphogenesis of the CJ, however, we come to a slightly modified concept of chamber ballooning that emphasizes the bilaterally paired origin of the heart. In the preceding sections of our review article, we have shown that the formation and remodeling of the CJ run in bilaterally paired patterns. The CJ of the primary ventricular tube has a non-uniform spatial distribution that reflects its origin from the left- and right-sided heart fields. The endocardial cushions/ridges are bilaterally paired structures, and the outgrowth of endocardial sprouts/projections into the CJ of the outer curvature of the ventricular bend runs in a bilaterally paired pattern. We have, furthermore, shown that the latter process does not stop at the basal surface of the myocardium. The endocardial sprouts/projections of the trabecular shields invade the myocardial wall and contribute to trabeculation and ventricular chamber ballooning. It is, therefore, tempting to speculate that the latter two processes might start in a bilaterally paired fashion. During a recent project on heart looping morphogenesis, which was conducted in the lab of one of us (J.M.), we found support for this idea. Scanning electron microscopic analyses of the outer shape changes of early embryonic mouse hearts have shown that the initial step of externally visible ventricular chamber ballooning is indeed characterized by the emergence of two half-balloons that expand toward the original ventro-lateral walls of the primary ventricular tube. The ventral wall of the outer curvature of the ventricular bend does not significantly participate in this early step of chamber ballooning (Figure 16A). With continuing expansion of the paired half-balloons, however, the ventral wall of the outer curvature becomes successively incorporated into the ballooning process so that the initially separated ballooning centers eventually merge to a single unit (Figure 16B,C).

The initially paired character of the emergence of externally visible embryonic heart chambers seems to correspond to the spatial patterns of myocardial proliferative activity that have been documented during early chamber formation of chick and human embryonic hearts (see Figure 7B in [119], and Figure 3H in [120]). 

In view of the observations described above, we have developed a modified concept of early heart chamber ballooning that distinguishes three primarily paired ballooning centers (Figure 16A_1_–C_1_). These three centers form at the level of the primary atrium, the level of the proximal portion of the ventricular bend, and the level of the distal portion of the ventricular bend. Each of these centers consists of one partner that derives from the left-sided heart field and another partner that derives from the right-sided heart field. The partners of the proximal and distal ventricular ballooning centers normally merge to form single units, which are called the embryonic left and right ventricle, respectively. These units give rise to the apical trabeculated regions of the prospective left and right ventricle, respectively. The partners of the atrial ballooning center remain separated and give rise to the atrial appendages of the prospective right and left atrium, respectively. We are aware of the fact that, at the present time, our modified ballooning concept is based exclusively on morphological data. Future studies are needed to see whether this concept is reflected by molecular expression patterns and in vivo tissue dynamics. 

## 6. Conclusions and Outlook

Our review has shown that the functional morphology of the CJ is a complex story, which affects many aspects of early heart development. Furthermore, it has become apparent that, at the present time, many of the above-mentioned concepts of the biomechanical relevance of the CJ have speculative characters and need support by experimental data. We do not think that it is a shortcoming of our article that we cannot present concepts that are supported by a multitude of experimental data. We rather think that this fact may stimulate future studies on early cardiac pump action. CJ-deficient embryos seem to be the model of choice for clarifying the significance of the CJ for the systolic and diastolic functions of the valveless embryonic heart. For such studies, we now can use imaging techniques, such as optical coherence tomography (OCT), which facilitate high resolution in vivo imaging of the solid and fluid dynamics of early embryonic heart action. The postulated roles of CJ-fibers in cardiac pump action and mechanosensing may stimulate renewed interest in uncovering the arrangement, molecular composition, and dynamics of CJ-fibers. Mathematical models may help to clarify the functional significance of elliptic and deltoid cross-sections of the ventricular tube.
